# Mimicking the Physicochemical Properties of the Cornea: A Low-Cost Approximation Using Highly Available Biopolymers

**DOI:** 10.3390/polym16081118

**Published:** 2024-04-17

**Authors:** Juan Hernández, Concepción Panadero-Medianero, Macarena S. Arrázola, Manuel Ahumada

**Affiliations:** 1 Centro de Nanotecnología Aplicada, Facultad de Ciencias, Ingeniería y Tecnología, Universidad Mayor, Camino La Pirámide 5750, Huechuraba 8580745, Santiago, Chile; juan.hernandeze@mayor.cl; 2 Centro de Biología Integrativa, Facultad de Ciencias, Ingeniería y Tecnología, Universidad Mayor, Camino La Pirámide 5750, Huechuraba 8580745, Santiago, Chile; maria.panadero@umayor.cl (C.P.-M.); macarena.arrazola@umayor.cl (M.S.A.); 3 Escuela de Biotecnología, Facultad de Ciencias, Ingeniería y Tecnología, Universidad Mayor, Camino La Pirámide 5750, Huechuraba 8580745, Santiago, Chile

**Keywords:** cornea, biomaterial, corneal-mimicking properties, physicochemical properties, chitosan, cytocompatibility

## Abstract

Corneal diseases represent a significant global health challenge, often resulting in blindness, for which penetrating keratoplasty is the clinical gold standard. However, in cases involving compromised ocular surfaces or graft failure, osteo-odonto keratoprosthesis (OOKP) emerges as a vital yet costly and complex alternative. Thus, there is an urgent need to introduce soft biomaterials that mimic the corneal tissue, considering its translation’s physicochemical, biological, and economic costs. This study introduces a cross-linked mixture of economically viable biomaterials, including gelatin, chitosan, and poly-D-lysine, that mimic corneal properties. The physicochemical evaluation of certain mixtures, specifically gelatin, chitosan, and poly-D-lysine cross-linked with 0.10% glutaraldehyde, demonstrates that properties such as swelling, optical transmittance, and thermal degradation are comparable to those of native corneas. Additionally, constructs fabricated with poly-D-lysine exhibit good cytocompatibility with fibroblasts at 72 h. These findings suggest that low-cost biopolymers, particularly those incorporating poly-D-lysine, mimic specific corneal characteristics and have the potential to foster fibroblast survival. While further studies are required to reach a final corneal-mimicking solution, this study contributes to positioning low-cost reagents as possible alternatives to develop biomaterials with physicochemical properties like those of the human cornea.

## 1. Introduction

The cornea is a fibrous and transparent tissue located at the sixth anterior part of the ocular globe and is mainly composed of water (78%) and collagen (17%). Its primary function is to bring two-thirds of the dioptric power to the eye, acting as a convergent lens [1]. Under either pathological or traumatism-derived corneal alterations, the vision can be negatively modified, promoting a reduction in the sensibility to contrast, a reduction in the visual field, a decrease in visual acuity, and, in extreme scenarios, total blindness [2,3]. Over 950 million people globally suffer from cornea-related visual impairments, with treatment costs reaching USD 7.8 billion in 2015 [4,5,6]. Depending on its etiology, corneal treatment, which often involves optical or surgical interventions, may escalate to keratoplasty (corneal transplantation) in complex cases [7,8]. However, the scarcity of corneal donors limits accessibility, implying that most patients do not have access to this tissue, leading to blindness [9]. Therefore, there is an urgent need to search for alternatives, particularly in the materials science and tissue-engineering fields. 

Among the alternatives at the clinic, keratoprostheses or artificial corneal implants are viable options to recover patients’ vision when no corneal donor tissue or treatment is available [10,11,12]. There are two main types: osteo-odonto-keratoprosthesis (OOKP) and modified osteo-odonto keratoprosthesis (MOOKP); in both cases, autologous tissue in combination with polymeric materials and complex surgeries are required. Regarding their outcomes, OOKP preserved its integrity in 94% of patients who finished their follow-up period, with an improvement in visual acuity better than 0.05 logMar (considering 51% of evaluated patients), and stabilized at the 10-year post-OOKP follow-up; yet, excluding cataracts, acquired glaucoma emerges as the most common side effect, with a prevalence of 36% after ten years [13]. MOOKP showed similar results, with a visual acuity improvement of 0.05 logMar (or better) in 78% of the patients after 37 months, with a success rate of 88.25% after the conclusion of the follow-up period [11]. While both techniques presented specific challenges, the overarching findings underscore the efficacy and potential of these procedures in enhancing the visual quality.

Corneal replacement using synthetic soft biomaterials has also been considered a viable option to solve the cornea donor shortage, with some biomaterials already in the advanced steps of clinical trials or on the market [14,15]. In this category, materials such as hydrogels [16], sponges [17], films [18], and polymeric networks [19] have been proposed, where commonly the main target acts as a stromal replacement. Regarding starting reagents, collagen from different sources [20], gelatin [21,22], silk [23], peptides [24], decellularized corneas [25,26], and self-assembly cellular systems [27], among others, have been employed to reach such an objective. For instance, Fagerholm et al. (2009) developed a synthetic corneal implant utilizing a form of engineered type III collagen cross-linked with 1-Ethyl-3-(3-dimethylaminopropyl)carbodiimide (EDC) and N-hydroxysuccinimide (NHS), which has already undergone clinical evaluation. In this work, the trial involving the lamellar graft was carried out on a cohort of 10 patients. Observations over 24 months revealed encouraging signs of epithelial regeneration, stromal cell proliferation within the implant, and notable nerve development in the vicinity [14]. Also, Jangamreddy et al. (2018), employing collagen-like-peptides conjugated with polyethylene glycol, made implants that were grafted into miniature-pig corneas to study their effects on corneal regeneration; their results suggested that the implant acts as a promotor of corneal tissue regeneration [24]. While the results are encouraging, besides inefficiency under certain conditions or undesired side effects, the most promising synthetic corneas require expensive reagents, such as recombinant human collagen or peptides, making it impossible for people from middle- and/or low-income countries to acquire them [28,29]. Thus, developing corneal substitutes using low-cost reagents is a pivotal task to reach the translation to a broad public. 

This work explores different hydrogel formulations using low-cost reagents and focuses their experimental assessment on the relevant parameters intended for corneal regeneration [30], including but not limited to optical, thermal, and environmental degradation, enzymatic degradation, cell cytotoxicity, and the cell adhesion properties. The results are compared with previous results obtained for native corneas and discussed as potential formulations, in terms of the physicochemical properties, to be employed in the generation of synthetic corneal implants.

## 2. Materials and Methods

### 2.1. Materials

Porcine skin gelatin type A (G1890), chitosan (448877), glutaraldehyde (G6257), acetic acid (A6283), lysozyme from chicken egg white (L6876), and phosphate-buffered saline tablets (P4417) were purchased from Sigma-Aldrich (St. Louis, MO, USA). Poly-D-lysine (A38904-01) was acquired from ThermoFisher (Waltham, MA, USA). Reagents were used as received. All the dissolutions were prepared using MilliQ water obtained from an Adrona CNB1901 MilliQ Ultrapure water purification system. 

### 2.2. Hydrogel Preparation

Different formulations of hydrogels were prepared considering the use of the following mixtures: gelatin (1% or 2% *w*/*v*), poly-D-lysine (1% *v*/*v*), and/or chitosan (1% *w*/*v*) dissolved in 0.5 M acetic acid at 40 °C under continuous stirring overnight. The selection of the proposed reagents was made based on their low costs and on previously described biomedical applications [31,32,33,34]. To form a three-dimensional network, each mixture was chemically cross-linked with glutaraldehyde in different concentrations (0.02, 0.05, and 0.10% *v*/*v*) following protocols previously reported by Farris et al. (2010), with some modifications [35]. Briefly, while the homogeneous solutions were still stirring, diluted glutaraldehyde was added dropwise, and the mixture was maintained at 40 °C for 24 h. The hydrogels were thoroughly washed three times to remove any possible excess of unreacted glutaraldehyde. Table S1 displays all the 18 formulations developed. After being cross-linked, the hydrogels were placed into molds of equal volumes and dried in a vacuum oven for 24 h at 40 °C to obtain dry and thin films. 

### 2.3. Hydrogel-Swelling Determination

The swelling of the hydrogel was calculated using Equation (1):

(1) $$\%\;Swelling=\frac{\left(m_{s}-m_{d}\right)}{m_{d}}\times 100$$

Here, *% Swelling* represents the percentage of swelling in the hydrogel, *m_s_* refers to the mass of the swollen material, and *m_d_* is the mass of the dry hydrogel. The dry mass was determined after incubating the material overnight at 40 °C in a vacuum oven. Conversely, the swollen mass was measured after immersing the hydrogels in water, removing them from the buffer solution, and gently blotting them to eliminate excess water. 

### 2.4. Characterization

#### 2.4.1. Fourier Transform Infrared (FTIR) Spectroscopy

FTIR spectroscopy was performed to identify the molecular structure using a Spectrum Two FTIR (Perkin Elmer, Waltham, MA, USA). Samples were dried in a vacuum oven before analysis. Measurements were conducted using an attenuated total reflectance (ATR) accessory, with 64 scans performed per sample. 

#### 2.4.2. Simultaneous Thermal Analysis (STA)

Simultaneous thermal analysis was performed using an STA 8000 (Perkin Elmer, Waltham, MA, USA), with measurements ranging from 20 °C to 500 °C. A scanning rate of 20 °C/min and a 20 mL/min nitrogen flux were applied as carriers. Measurements were carried out for swollen and dry hydrogels, with starting reagents serving as control samples.

#### 2.4.3. Optical Properties

Hydrogels were analyzed in a UV-Vis spectrophotometer Spectroquant Prove 300 (Merck, Darmstadt, Germany) using the transmittance mode spanning from 190 to 900 nm to assess the light transmittance. Refractive index measurements were also conducted using a liquid refractometer (Soonda, Beijing, China).

#### 2.4.4. pH and Enzymatic Degradation

Hydrogel stability and degradation were examined at pH values of 4, 7, and 10, utilizing 2-(N-morpholino)ethanesulfonic acid (MES), phosphate-buffered saline (PBS), and tris(hydroxymethyl)aminomethane (Tris) buffers at 10 mM, respectively. In each case, the pH was adjusted by the dropwise incorporation of HCl 0.1 M to reduce it or of NaOH 0.1 M to increment it. Known hydrogel masses were incubated with the respective buffers at 37 °C, and mass evaluations were conducted at various time intervals. The percentage mass loss (% *m_L_*) was calculated as follows:

(2) $$\%\;m_{L}=\left(\frac{m_{0}-m_{x}}{m_{0}}\right)\times 100$$

Here, *m_x_* denotes the hydrogel mass at a specific time, and *m*_0_ represents the initial mass. For enzymatic assessment, lysozyme at 55 units/μL was prepared in PBS (pH 7.4) and utilized following previously outlined methodologies.

### 2.5. Cytocompatibility Evaluation

Mouse embryonic fibroblast NIH/3T3 cells (ATCC, CRL-1658) were seeded on 12-well plates containing the hydrogels, with a density of 35,000 cells/cm^2^, in Dulbecco’s Modified Eagle Medium (DMEM) (Gibco, Waltham, MA, USA, 12800-017), supplemented with 10% fetal bovine serum (SFB) (Capricorn Scientific, Edsdorfergrund, Germany, FBS-HI-11A) and 1% penicillin G–streptomycin (Gibco 15140-122) at 37 °C under a humid atmosphere at 5% CO_2_. As a control, a well with cells seeded directly on the plate, without a hydrogel, was included. Bright-field microscopy was employed to follow the cell proliferation, and representative images were collected at 24, 48, and 72 h. The non-adherent cells to the hydrogels were removed by gently rinsing them with sterile PBS (pH 7.4). After 72 h, cells were stained with DAPI (1 µg/mL) for nucleus detection and assessed for viability using a DMi8 Leica Fluorescent Microscope.

Plots and statistical analysis were performed with GraphPad Prism software v8.0, employing the results obtained from n = 3 independent experiments.

## 3. Results and Discussion

### 3.1. Reagent Selection and Hydrogel Preparation

Materials intended for corneal regeneration must fulfill several conditions, such as water retention, transparency, an appropriate refractive index, degradability, and biocompatibility [19,31]. While these are relevant for biomaterial development, selecting reagents that can allow the synthetic cornea to be obtained is equally relevant, considering both the applicability and accessibility for future translation into the clinic. In this work, gelatin (G), chitosan (C), and poly-D-lysine (P) were chosen not only due to their previously described properties and applications in biomaterial development [31,32,33,34], but also considering their low market price. Particularly, in the case of gelatin selection, it is one of the gold-standard reagents in tissue engineering, as it provides desirable biocompatibility, biodegradability, and cell adhesion, allows enzymatic degradation without an immunogenic response, and is cost-effective, to highlight some characteristics [36]. Chitosan was chosen because it is a highly available biopolymer that is, additionally, biocompatible; it has excellent biodegradability, providing matrix support, which differentiates it from other natural polysaccharides, and it has also been described as bioactive against bacteria [37]. Finally, regarding the selection of poly-D-lysine, this is a cationic polyaminoacid that has been recently explored in a more profound manner for tissue engineering, showing good biocompatibility and degradability and promoting cell adhesion, survival, and growth, ascribed to their positive charge [38,39]. 

Then, to establish the hydrogel formulations, different mixtures containing gelatin (G), chitosan (C), and poly-D-lysine (P) were used (Table S1). All cross-linked hydrogels developed were homogeneous solutions before drying in a vacuum oven. After this drying step, each hydrogel was collected (as a thin film) for the next steps. 

### 3.2. Hydrogel Swelling

**Figure 1:**
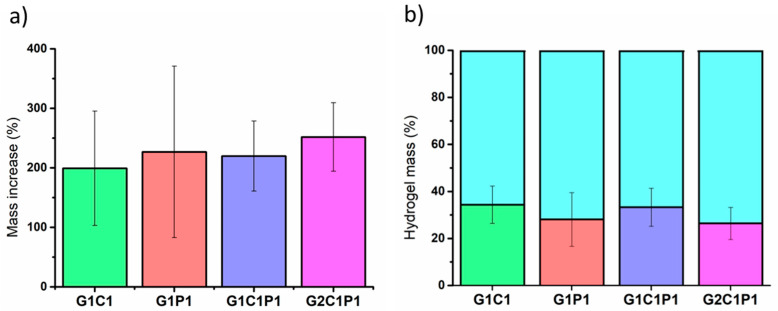
Selected hydrogel formulations’ swelling. (**a**) The swelling-percentage bar plot. (**b**) Hydrogels’ mass content percentages established based on their dry (multicolor bars) and water (cyan-colored bars) contents. Measurements were performed considering n = 3. Values represent the mean ± SEM. Significance was analyzed by RM-ANOVA and Tukey’s test for multiple comparisons, resulting in no significance.

The water content and its relationship with the dry mass are relevant parameters for mimicking corneal tissue. For instance, optical transparency depends on the water content, as there is a balance with the passive water transport mechanism [40]. Further, the swelling can determine its ability to avoid osmotic gradients, which might result in a decline in the clarity of vision for transparency loss [41]. Thus, the swelling was evaluated considering the 18 formulations exposed in Table S1. Figure 1a presents the four formulations with the closest reported swelling percentages to the reference corneal values [1]. These four selected formulations contained 0.10% *v*/*v* glutaraldehyde (GA), which was employed to promote the covalent cross-link. In addition, Figure 1b shows the mass percentage values related to the water and dried-mass contents of the same formulations. The remaining 14 formulations were not posteriorly considered for the following assays, as they did not meet the required swelling-percentage criteria. The results are presented in Figure S1. 

Regarding the established formulations, particularly those presented in Figure 1, the observed swelling and total mass contents are closely related to those reported for native corneas, which have been described as having from around 22 to 34% dry mass with respect to the total mass [1]. Further, the results presented in Figure S1 show that these formulations did not match the previously mentioned average dry mass; thus, they were not selected to continue in the following stages. As stated, water retention is fundamental for conserving the transparency, ocular pressure, and mechanical properties [41]. Similarly, other authors have also established the relevance of swelling for developing soft corneal biomaterials, emphasizing the importance of optimizing the mechanical properties of these biomaterials [42], as these properties are crucial for withstanding ocular pressures and ensuring long-term stability and functionality.

### 3.3. Hydrogel FTIR Molecular Analysis

**Figure 2:**
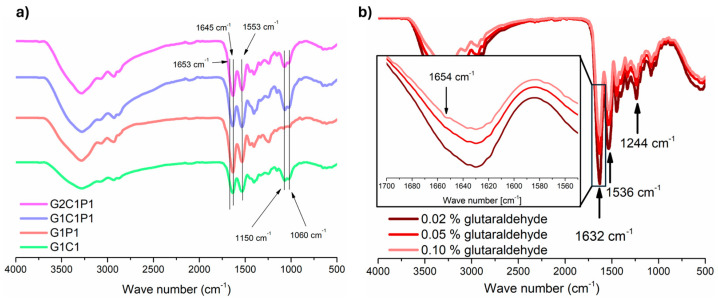
Infrared spectroscopy of selected biomaterial formulations. (**a**) FTIR spectra of four formulations; arrows indicate those bands associated with the amine groups. (**b**) FTIR spectra of formulation G1P1 utilizing increasing concentrations of GA; inset shows a zoom to the zone where the imine signal can be appreciated.

Once the four candidates’ formulations were established, and because they share the same GA percentage, they were referred to as G1C1, G1P1, G1C1P1, and G2C1P1, with the numbers referring to its concentrations in volume/volume percentages. For these formulations, a physicochemical characterization was performed, starting with the molecular analysis employing FTIR spectroscopy, which allows for the identification and structural retention of the starting reagents, as well as for identifying the cross-linking between the reagents to guarantee long-term stability when compared to formulations without cross-linking. Focus was placed on the amine groups, which actively participate in the GA cross-linking reaction. Figure 2a shows the obtained FTIR spectrum for each formulation after cross-linking with GA. It can be observed that most of the characteristic bands corresponding to the starting reagents are retained (Figure S2). Along this line, Figure 2a shows that the selected formulations retain the characteristic peaks of the starting reagents (Figure S2), such as the N-H stretching at 3285 cm^−1^ with a broadness enhancement due to the presence of O-H groups observed within the same range (3500–3000 cm^−1^), and carbonyl (C=O) presence at 1632 cm^−1^, with the presence of the formation of the amide bond (C-N) observed at 1553 cm^−1^. Further, only the formulations containing chitosan have characteristic peaks that can be observed at 1150 and 1060 cm^−1^, corresponding to the presence of a saccharide and C=O stretching [43]. Yet, to corroborate the successful cross-linking of the material, increasing GA concentrations were incorporated into the formulations and posteriorly evaluated by FTIR (Figure 2b). Here, it is possible to observe that increasing the concentration of GA decreases the peak at 1653 cm^−1^, which can be associated with imine formation [35,44,45]. 

### 3.4. Hydrogel Optical Properties

**Figure 3:**
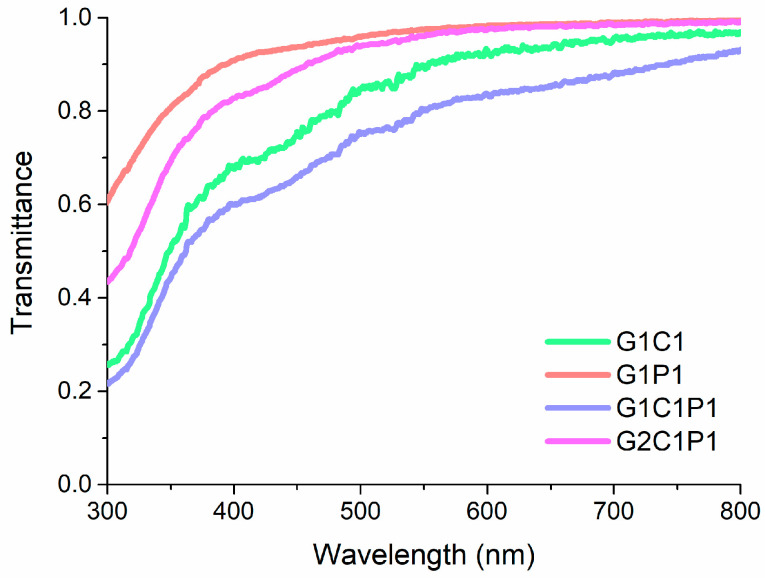
Selected formulations’ UV-Vis transmittance spectra.

Optical properties are fundamental for biomaterials with corneal applications, particularly considering the transmittance as a critical parameter for materials intended for corneal replacement. In this regard, Figure 3 shows the main results obtained for the four formulations, for which there is a low percentage of transmittance below 300 nm and a sustained increase until approximately 500 nm, reaching a ~80% transmittance. At longer wavelengths, an almost full transmittance is observed for all the formulations. Thus, the results presented in Figure 3 are comparable to the previously described experimental results by Botettner and Wolter for a native cornea [46] and those estimated by Peris-Martinez et al. [47], where it can be observed that there is a quick increment in the light transmitted between 300 and 500 nm (reaching around 70%) and, from there, a transmittance increase up to 98%. 

The refractive index is another key optical parameter for materials intended for corneal tissue replacement. This parameter plays a significant role in ensuring correct image formation on the retina. Table 1 shows the refraction index values obtained for all the formulations, ranging from 1.336 to 1.339, which is within the range of the reported corneal values (1.335–1.439) [48]. Yet, measuring the corneal native refractive index depends on the considered structures (e.g., cornea plus tear film usually shows values close to 1.4) [48]. Thus, while the presented values are in the lower range, it should be considered that these can be adjusted by considering their posterior integration within the ocular tissue.

### 3.5. Hydrogel Thermal Stability Analysis

**Figure 4:**
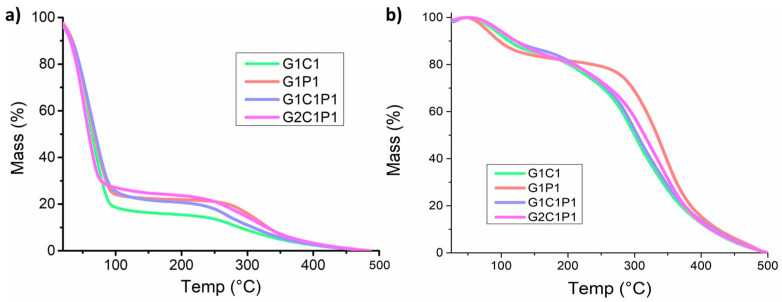
Thermal analysis of the selected formulations. (**a**) Thermogravimetric behavior of the hydrated hydrogel formulations. (**b**) Thermogravimetric behavior of the dehydrated samples.

Thermal analysis was performed to provide information not only about the thermal stability but also related to the composition. To evaluate this, two approximations were made: one with the swelled hydrogel and another with the dry material (Figure 4). Regarding the swollen hydrogels, Figure 4a shows the thermal gravimetric analysis (TGA) performed on the formulations considering a temperature range from 20 to 500 °C, where around 75% of the weight loss is observed at ~100 °C, and another important weight loss is observed at temperatures close to 300 °C. Similarly, Figure 4b shows the results obtained for the dried hydrogels, for which a less pronounced weight loss is observed before 100 °C (~20%) and a more marked loss is observed at temperatures close to 300 °C. The mass loss observed at temperatures near 100 °C can be ascribed to the water loss [49]. Further, these results coincide with those previously exposed in the section called Hydrogel Swelling (Section 3.2), demonstrating that the proposed formulations have swelling properties close to those of a native cornea. Similarly, the thermal analysis of the previously dehydrated samples shows a similar thermal behavior to that of the hydrated sample, ascribed to the high thermal stability. Further, Figure S4 show the obtained differential scanning calorimetry (DSC) results, employed to perform a more detailed analysis of the possible phase changes at temperatures between 15 and 60 °C. However, no signals were recorded within the selected range, indicating the absence of thermal changes since thermal stability was indicated [50]. Regarding the degradation process itself, it has been observed that between 200 and 240 °C (depending on the formulation), the mass weight loss can be related to the detachment of lateral groups from the main molecule chains [51] to continue similar degradation behavior posteriorly. The data collected for the starting reagents are presented in Figure S3. 

### 3.6. Hydrogel Degradation

**Figure 5:**
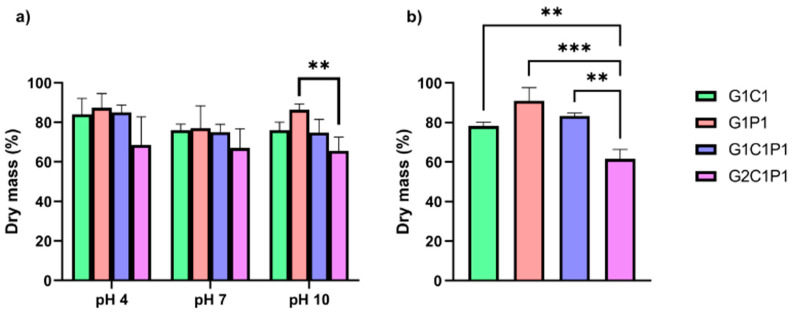
Hydrogel formulation degradation. (**a**) Hydrolytic degradation prompted by pH action; measurements were taken at pH 4, 7, and 10. (**b**) Lysozyme enzymatic degradation for the selected formulations (in PBS, pH 7.4). Significance was analyzed using RM-ANOVA and Tukey’s test for multiple comparisons. ** *p* < 0.01, *** *p* < 0.005.

Degradation plays another relevant role in the design of biomaterials for corneal regeneration, as these should be degraded at a rhythm that adjusts to the regeneration of the corneal tissue because, in case of a fast degradation rate, there will be a quick loss of dimensional stability. If such a rate is slow, it will not allow tissue replacement or will promote compromised tissue with uneven regeneration [52]. This work evaluated two parameters: the pH and enzymatic degradation, both presented in Figure 5. Regarding the pH, hydrolytic degradation is a common degradation mechanism in which water can impulse the scission of molecules into smaller ones, where pH plays a key role [53]. Considering the cornea, the main interest is at pH 7, as the average pH value of tears is 7 [54], yet stability assays were also performed under acidic and basic conditions to evaluate a broader range. Figure 5a shows that, after three weeks, all the tested formulations lost mass weight (yet retained at least two-thirds of their initial mass) at all the considered pH values. Still, no significant differences were observed among the formulations at different pH levels. Under normal conditions, the corneal stroma has a neutral pH, but variations in the pH can provoke alterations in its morphology and transparency [55,56]. Thus, designing biomaterials with superior stability should avoid such modifications. As per the results, at neutral pH, there is a retention close to 75% of the mass weight after three weeks. Still, further work should be explored for additional time. Two highly described enzymes can degrade materials found in the cornea: collagenase and lysozyme [57,58]. The first are enzymes that degrade the collagen into smaller peptides by breaking the peptide bonds, commonly associated with tissue damage [58]. In contrast, lysozyme can be found in tears, and it is responsible for the bacterial cell membrane degradation of some strains via the breaking of their glycosidic bonds [59], which has also been described for chitosan [60,61]. In this work, the degradation action of lysozyme was tested for the formulations utilizing enzyme concentrations close to those reported for tears in healthy subjects [62]. Figure 5b shows that, after a two-week evaluation, the formulations could keep over 50% of their mass weights, whereas those incorporating chitosan showed the quickest degradation. It is also possible to corroborate, as previously stated, that lysozyme cannot promote the degradation of gelatin and PDL. Regarding collagenase action, this was not evaluated, considering that a previous work by Romo-Valera et al. (2021) compared the degradation action of collagenase over cross-linked collagen films vs. gelatin equivalents, demonstrating that the gelatin showed higher resistance toward enzymatic degradation by collagenase, retaining mass percentages close to 80% after 24 h [63]; still, future work should also explore its action at longer exposure times. Yet, a relevant aspect of biomaterial degradation corresponds to the degradation rate and applicability of the biomaterials in real situations. In general terms, there are two general answers. For biomaterials intended for replacement, degradation is not desired, as it decreases the lifetime usability of any potential solution. For biomaterials designed for tissue regeneration, the required time will depend on the cell migration and extracellular matrix (ECM) regeneration, which should provide the desired regeneration [19]. The latter is probably the most ambiguous question, as the time required will depend on several factors, including the subject being treated. Nonetheless, as initially stated, both fast and slow rates are undesired, extreme scenarios [52]. For example, a previous study established that if cells are seeded before transplantation, a two-week degradation rate that can be guaranteed can be optimal for corneal regeneration [64]. Yet, it should be considered that, generally, in experimental terms, degradation is only viewed as a function of the time exposition to the physiological temperature and/or pH variations evaluating changes in the mass, which does not consider other relevant aspects, such as enzymatic degradation. Still, in this work, the previously mentioned conditions were tested, showing pH and thermal stability for up to three weeks, conserving 80% of the weights of the hydrogels, and, at two weeks, conserving more than 75% of the weights of the materials when exposed to lysozyme. However, while these results align with previous studies, they should be carefully evaluated, as the degradation rate depends not only on in vitro parameters but also mainly on in vivo behavior. 

### 3.7. Hydrogel Cytocompatibility

**Figure 6:**
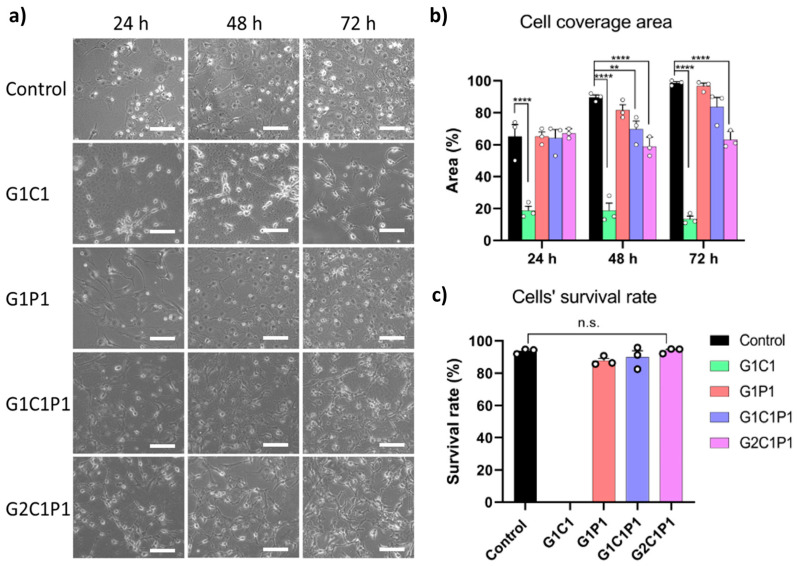
NIH/3T3 fibroblast cytocompatibility when exposed to the hydrogel formulations at different times. (**a**) Representative bright-field microscopy images at different time points (scale bars: 150 µm). (**b**) Cell coverage areas in percentages at different time points. (**c**) Survival rate percentages at 72 h. Values represent the mean ± SEM from n = 3. Significance was analyzed using RM-ANOVA and Tukey’s test for multiple comparisons. ** *p* < 0.01, **** *p* < 0.001, n.s. non-significant.

While biocompatibility evaluation comprises several biological parameters, cytocompatibility is one of the easiest to assess, as it can provide information about the cellular toxicity and adhesion, which are both important for tissue regeneration [63]. Embryonic fibroblast cells have been much described in the literature for studies on the cytocompatibility and cell adhesion in materials [65]; thus, they were chosen to evaluate the formulation performances in vitro. Further, fibroblasts are a differentiated cellular type of keratocytes in the stroma, and they participate in the immune system, differentiation, and corneal wound healing [66]. Hence, in vitro assays were performed by seeding embryonic fibroblasts on top of the hydrogels to evaluate their cytocompatibility at different time points (see Figure 6). 

Figure 6a shows representative bright-field images for all the formulations. The cells were able to adhere to the hydrogel surface and proliferate at rates comparable to the control at all the times tested for the G1P1, G1C1P1, and G2C1P1 formulations. Yet, for the G1C1 formulation, cells were not able to fully adhere to the hydrogel. Further, Figure 6b presents the percentages of the cell coverage areas at different times, where only the G1P1 formulation showed cell density values comparable to those of the control. Finally, Figure 6c shows the percentage of cell survival after 72 h exposure to the hydrogel formulations, showing that the values for all the formulations are comparable to those of the control, except for G1C1, for which the survival rate was impossible to determine due to the lack of cell adhesion with this formulation, which does not match the high cell adhesion on the remaining formulations (Figure S5). Such an effect can be ascribed to the absence of poly-D-lysine in this formulation, as this reagent has been described as a cellular adhesion promoter because of the favored electrostatic interactions between the positive-charge lysine residues and negative-charge groups present in the cellular membrane [67,68,69]. Despite this, formulations G1P1, G1C1P1, and G2C1P1 had similar covered surface percentages at 24 h but differentiated significantly at longer times. Along this line, formulation G1P1 promotes a proliferation behavior like that of the control, which can be justified by the absence of chitosan within the formulation. Further, these three formulations showed similar survival rate percentages after 72 h.

Previously, Ratanavaraporn et al. (2006) compared the cytocompatibility of fibroblasts on collagen and gelatin scaffolds, cross-linked by employing a thermal dehydration process [70]. They found that both formulations showed similar physicochemical and biocompatible behaviors. However, the gelatin showed a quicker degradation rate, requiring a more robust cross-link method and the presence of additional reagents. In another work, Romo-Valera et al. (2021) seeded epithelial and fibroblast cells onto soy protein isolate, collagen, and gelatin scaffolds cross-linked with lactose, determining that, after 72 h, the gelatin showed higher cell viability than the collagen scaffold for both cellular lines and improved the optical properties [63]. Another relevant point regarding the possible toxicities derived from the hydrogel formulations comes from the use of glutaraldehyde, which has been previously described as a toxic agent [71]. GA is a dialdehyde molecule that allows the cross-linking between functional groups (such as carbonyl or amine groups), acting as a spacer between them. Its use in biomedicine has been extended since at least 1980 [72]. Yet, it is true that concentrations over 2.5% *v*/*v* have been described as toxic when in direct contact with cells or tissue. However, below this limit, the mentioned toxicity tends to be from low to none as the concentration decreases [73]. Further, the current preparation of materials allows for the removal of the unreacted GA by adding quenchers (such as glycine) and/or by washing the excess GA [74]. This work employed GA concentrations up to 0.1%, which, coincidently with previous reports, did not show any toxicity in the in vitro test. However, further studies should be performed to unravel not only GA’s possible cytotoxicity but also other possible effects of the final material. 

## 4. Conclusions

In this study, formulations incorporating gelatin, chitosan, and poly-D-lysine exhibited promising attributes for corneal regeneration, including optimal swelling behavior, molecular stability, as evidenced by FTIR spectroscopy, and essential optical properties, all of them closely related to those previously reported for native corneas. Notably, incorporating poly-D-lysine significantly improved the cytocompatibility, suggesting its pivotal role in enhancing cellular interactions that are vital for tissue integration and regeneration. While these formulations show substantial promise, addressing challenges related to differential degradation is crucial. Future directions of this work should look for its usage as a potential bioink for 3D bioprinting corneal applications. Continued refinement in these areas will likely propel these formulations closer to clinical translation, offering more accessible, effective, and enduring solutions for corneal regeneration.

## Figures and Tables

**Table 1:** Refraction index values of the selected formulations. Measurements were carried out at room temperature. Native-cornea-reported values are also presented for comparison.

Formulation	Refractive Index (SD)
G1C1	1.33759 (0.00029)
G1P1	1.33610 (0.00133)
G1Q1P1	1.33751 (0.00070
G2C1P1	1.33900 (0.00065)
Cornea ^a^	1.335–1.432

^a^ Data extracted from reference [48].

## Data Availability

The raw data supporting the conclusions of this article will be made available by the authors upon request.

## Supplementary Materials

The following supporting information can be downloaded at: https://www.mdpi.com/article/10.3390/polym16081118/s1, Table S1: Initial established formulation combinations for hydrogel preparation; Figure S1: Swelling of non-selected formulations; Figure S2: FTIR spectra of formulations’ precursors; Figure S3: Thermogravimetric analysis of formulations’ precursors; Figure S4: DSC of selected formulations; Figure S5: Representative microscopy images of NIH/3T3 fibroblasts stained with DAPI.
