# Supplementary material for: Mimicking the Physicochemical Properties of the Cornea: A Low-Cost Approximation Using Highly Available Biopolymers
Source: Polymers (Basel). 2024 Apr 17;16(8):1118. doi: 10.3390/polym16081118 (PMC11053614; doi:10.3390/polym16081118)
Supplement: Supplementary file 1 [file polymers-16-01118-s001.zip › polymers-2894357-supplementary.pdf]

Supplementary Material

Page		Description
S1	………………………………………………………………..	This page
S2	………………………………………………………………..	Table S1
S3	………………………………………………………………..	Figure S1
S4	………………………………………………………………..	Figure S2
S5	………………………………………………………………..	Figure S3
S6	………………………………………………………………..	Figure S4
S6	………………………………………………………………..	Figure S5

Table S1. Initial established formulation combinations for hydrogel preparation.

Formulation number	Gelatin (G) %	Chitosan (C) %	Poly-D-Lysine (P) %	Glutaraldehyde %
1	1.0	1.0	0.0	0.02
2	1.0	1.0	0.0	0.05
3	1.0	1.0	0.0	0.00
4	1.0	0.0	1.0	0.02
5	1.0	0.0	1.0	0.05
6	1.0	0.0	1.0	0.00
7	1.0	1.0	1.0	0.02
8	1.0	1.0	1.0	0.05
9	1.0	1.0	1.0	0.00
10	2.0	1.0	0.0	0.02
11	2.0	1.0	0.0	0.05
12	2.0	1.0	0.0	0.00
13	2.0	0.0	1.0	0.02
14	2.0	0.0	1.0	0.05
15	2.0	0.0	1.0	0.00
16	2.0	1.0	1.0	0.02
17	2.0	1.0	1.0	0.05
18	2.0	1.0	1.0	0.00

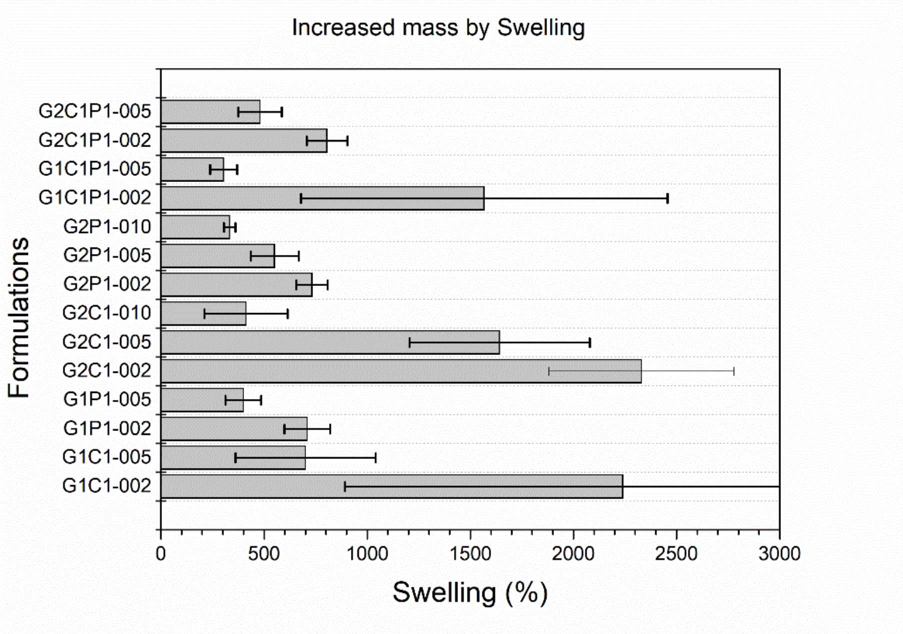

Figure S1. Swelling of non-selected formulations.

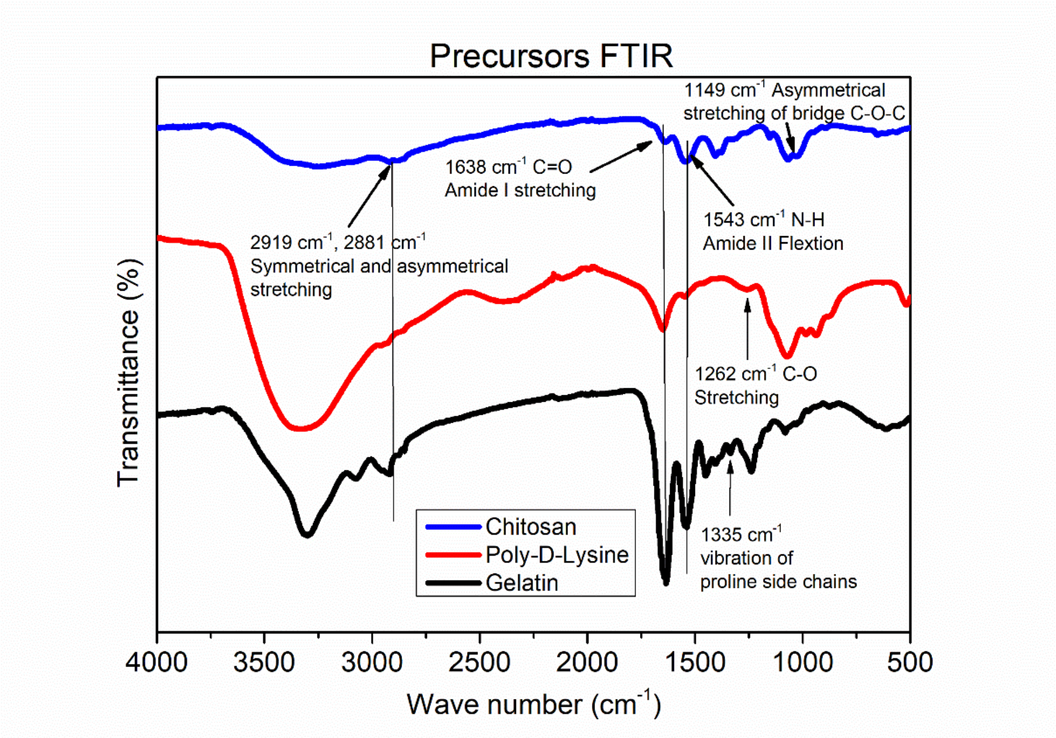

Figure S2. FTIR spectra of formulations´ precursors.

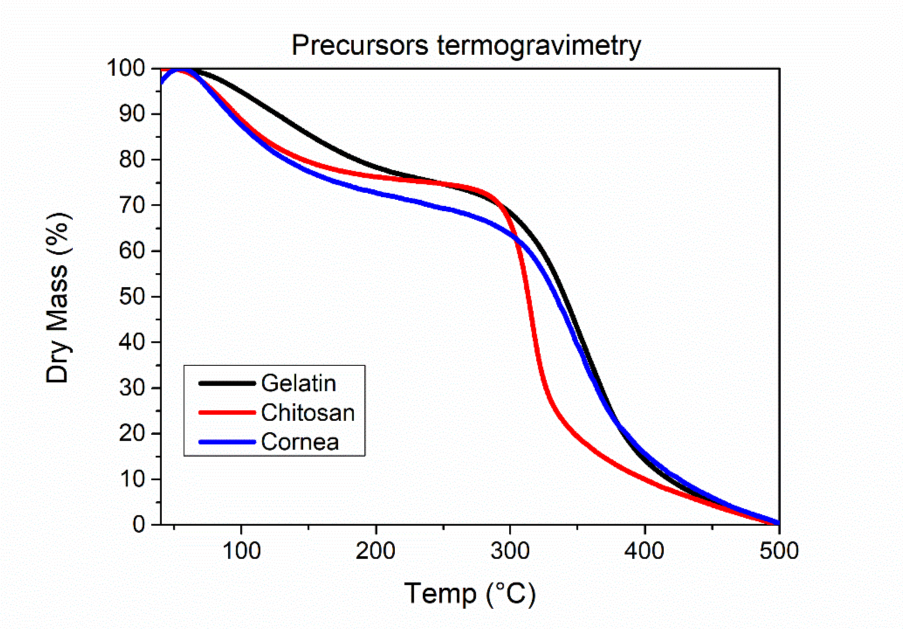

Figure S3. Thermogravimetric analysis of formulations´ precursors.

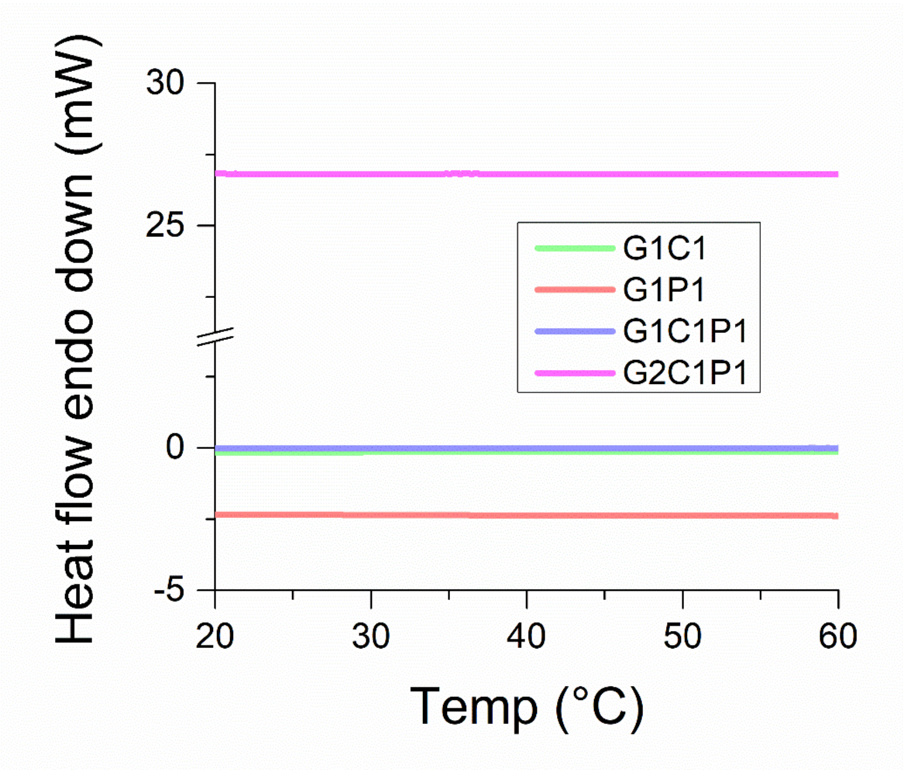

Figure S4. DSC results were obtained for the samples considering the range between 15 and 60 °C.

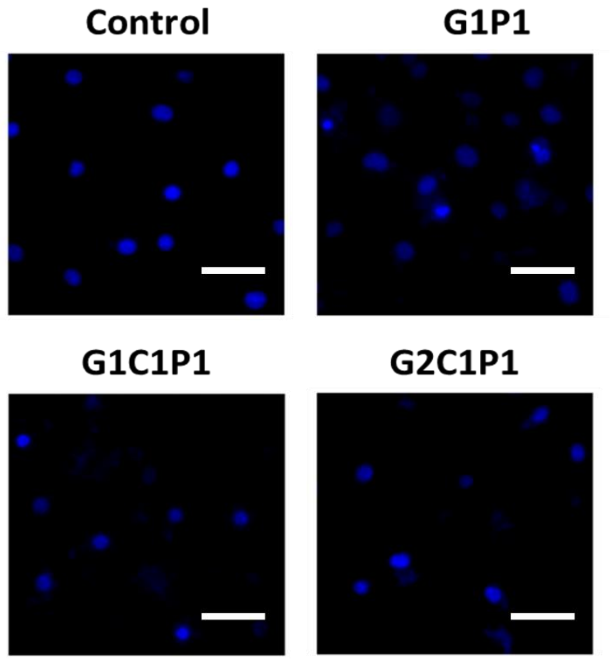

Figure S5. Representative microscopy images of NIH/3T3 fibroblasts stained with DAPI after 72 h exposure to the hydrogel formulations. Scale bars: 150 µm.
